# Nano-size uni-lamellar lipodisq improved *in situ* auto-phosphorylation analysis of *E. coli* tyrosine kinase using ^19^F nuclear magnetic resonance

**DOI:** 10.1007/s13238-014-0129-x

**Published:** 2015-01-07

**Authors:** Dong Li, Juan Li, Yonglong Zhuang, Longhua Zhang, Ying Xiong, Pan Shi, Changlin Tian

**Affiliations:** 1 Hefei National Laboratory for Physical Science at the Microscale & School of Life Science, University of Science and Technology of China, Hefei, 230026 China; 2 High Magnetic Field Laboratory, Chinese Academy of Sciences, Hefei, 230031 China; 3 Department of Chemistry, Anhui University, Hefei, 230027 China

**Dear Editor,**

Among many of biophysical methods, ^19^F NMR spectroscopy has emerged as a powerful tool for characterizing protein structure, dynamics and function properties due to high intrinsic sensitivities of fluorine, 100% natural abundance of the NMR-active spin, the absence of any natural background in cells, and exquisite sensitivities of ^19^F chemical shift to environment (Shi et al., 2011; Shi et al., 2010). With evolved aminoacyl-tRNA synthase specifically recognizing the ^19^F containing unnatural amino acids, and suppressed amber stop codon by tRNA_CUA_, several ^19^F containing unnatural amino acids were incorporated into proteins in bacteria (Hammill et al., 2007; Jackson et al., 2007). Achievements of the protein-specific and site-specific ^19^F incorporation provided great opportunities for *in cell* or *in situ* protein structure, dynamic and function studies. Recently, ^19^F-tfmF (trifluoromethyl-phenyl alanine) was incorporated to diacyl-glycerol kinase (DAGK) and we have achieved DAGK’s conformation and dynamics analysis in native lipid bilayer environment using ^19^F solid state NMR directly through bacteria membrane separation, without laborious protein purification and lacking negative interferences from detergents (Shi et al., 2012). However, the lipid system separated from bacteria membrane spontaneously formed multi-lamellar vesicles (MLV) and high proportion numbers of membrane proteins located in inner vesicles, which provided physical barrier for accessibilities of soluble ligands or interaction partner proteins. The poly-styrene-maleic-acid (SMA) can wrap around lipid to form nanoparticles (with average size of 10 nm), leaving the lipid bilayer a disc shape (lipodisq) (Knowles et al., 2009). The monodispersed lipodisq was reported to preserve the integrity of transmembrane proteins and form biocompatible, thermostable and soluble nano-particles (Orwick-Rydmark et al. 2012; Orwick et al., 2012). Very importantly, the two sides of the lipodisq can be accessed by soluble compounds or partner proteins, which provided great potential and convenience for biophysical analysis of membrane proteins in a minimized lipid environment.

Tyrosine phosphorylation is a reversibly post-translational modification that regulates many aspects of cellular functions (Hunter, 2009; Johnson and White, 2012). Tyrosine phosphorylation can be activated in both auto- and cross-catalytic by kinases in the presence of ATP and Mg^2+^, while the phosphorylation can be removed by phosphatases (protein tyrosine phosphatase, PTP). *E. coli* tyrosine kinase (ETK) is a transmembrane protein containing two transmembrane helices and a soluble kinase catalytic domain (ETK-CD). The ETK-CD had the auto-phosphorylation site Tyr574 and a tyrosine rich C-terminal tail (Lee et al., 2008) (Fig. 1A). Structure and function studies illustrated that the phosphorylated Tyr-574 consequently enabled cross-phosphorylation of the C-terminal tyrosine rich tail of ETK or tyrosine residues in other substrate proteins (Lee et al., 2008).

**Figure 1:**
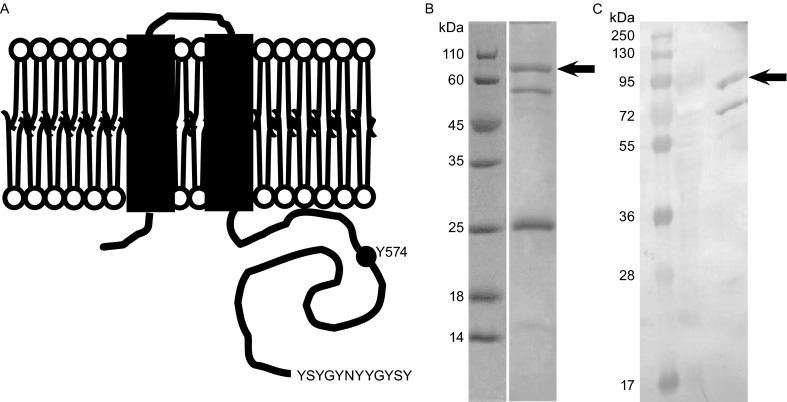
F2Y was incorporated at Tyr574 site of ETK-FL protein. (A) Topology diagram of an ETK protein in lipid bilayer, containing two transmembrane helices and a cytosolic tyrosine kinase domain including the auto-phosphorylation site Y574 and tyrosine rich C-terminal tail; (B) Coomassie blue staining of purified ETK-Y574F_2_Y; (C) Western-blot of purified ETK-Y574F_2_Y

Previously, we have developed genetically encoded unnatural amino acid (3,5-difluorotyrosine, F_2_Y) to implement site specific incorporation in ETK-CD (Li et al., 2013). Since the ^19^F spin was located neighboring to the active hydroxyl group, phosphorylation of the tyrosine immediately resulted in ^19^F chemical shift differences (i.e. −134.54 ppm for dephosporylated tyrosine and −122.30 ppm for phosphorylated tyrosine) (Li et al., 2013). However, the phosphorylation analysis of ETK-CD was implemented *in vitro* using purified proteins, while a crucial difference between *in vitro* and *in situ* conditions was expected. Especially, the *in vitro* sample contained rather high concentrations of macromolecules and lacked of native membrane environment (Barnes and Pielak, 2011). Here, phosphorylation property of Tyr574 in purified ETK-CD (Li et al., 2013) and full-length ETK (ETK-FL) in native *E. coli* membrane will be illuminated.

In this study, the F_2_Y was incorporated at Tyr574 site of ETK-FL protein through conventional unnatural amino acid incorporation methods. The ETK-FL protein with His_6_-tag in the N-terminus was expressed in *E. coli* membrane through expression condition optimizations. Brief protein purification was conducted using Ni-NTA affinity chromatography. Coomassie blue staining (Fig. 1B) and Western blot (Fig. 1C) analysis of the SDS-PAGE on partially purified proteins demonstrated the expressed ETK-Y574F_2_Y as 95 kDa (black arrow), while the band around 58 kDa was suspected as the truncated proteins stopping at amber stop codon introduced at the Tyr574 site, since the His_6_-tag was expressed at N-terminal of the ETK protein.

Retaining native membrane environment (without protein purification steps) is important for structure and function studies of the membrane proteins in their physiological condition. Here, the *E. coli* membrane fraction was obtained through ultracentrifugation method. Before the auto-phosphorylation analysis of ETK-FL with the presence of Mg^2+^ and ATP, the dephosphorylation treatment using PTP1B was implemented to remove any trace phosphorylations. Both the treatment by the PTP1B and Mg^2+^/ATP required direct interactions between the small protease or compounds and the cytosolic domain of ETK-FL. However, multi-lamellar vesicles (MLV) will be formed from bacteria membrane spontaneously (Fig. 2A). The MLV would not only have high proportions of ETK-FL locate in inner vesicles, but also have dual orientations of the ETK-FL in outer vesicle, leaving only half populations of ETK-FL with the exposure of its cytosolic domain. Therefore, the liposome MLV provided huge physical barrier for accessibilities of soluble ligands or interaction partner proteins for membrane protein studies. To overcome this hindrance effect of MLV, the conventional procedures was to mix the compounds or protein partners with the target membrane protein before reaction, with extensive liposome re-distribution procedures, i.e. several cycles of sonication-freeze-thaw. This procedure obviously requires complicate sample treatments, possibly having unexpected adverse influences to correct folding of proteins, but also makes it impossible to analyze kinetics of protein interactions. To improve accessibilities of soluble PTP1B or other compounds to ETK-FL in native lipid environment and also to simply the experimental procedure, poly-styrene-maleic-acid (SMA3000 3:1) compounds (purchased from Malvern Cosmeceutics company, UK and hydrated in authors’ laboratory) were applied to wrap up the lipid bilayers to form uni-lamellar lipodisq (Fig. 2A).

**Figure 2:**
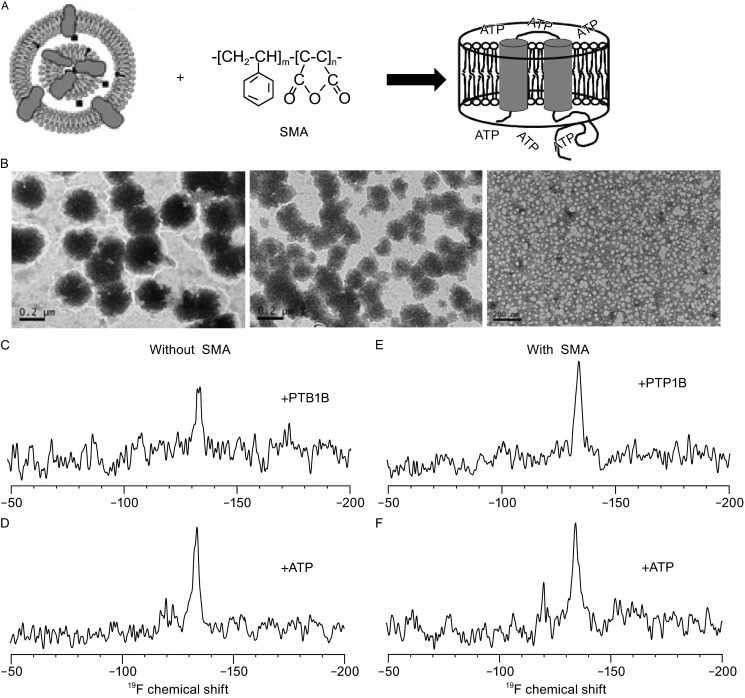
SMA wrapped uni-lamellar liposome elevated phosphorylation level of ETK-FL-Y574-F2Y. (A) Topology diagram of an ETK-FL in multi-lamellar lipid bilayer vesicles, composition of poly-SMA and topology diagram of an ETK protein in lipodisq; (B) Transmission electron microscopy pictures of native *E. coli* liposomes after freeze-thaw-bath sonication with addition of 0, 10, 30 μL poly-SMA3000 (3:1). The 0.2 μm bar was shown in the same size in the three pictures. (C) ^19^F NMR spectrum of ETK-Y574F_2_Y in native liposome with the presence of PTP1B; (D) ^19^F NMR spectrum of ETK-Y574F_2_Y in native liposome with the presence of ATP and Mg^2+^; (E) ^19^F NMR spectrum of ETK-Y574-F2Y in lipodisq with the presence of PTP1B; (F) ^19^F NMR spectrum of ETK-Y574F2Y in lipodisq with the presence of ATP and Mg^2+^

To achieve suitable size of lipodisq, the appropriate concentrations of SMA3000 to *E. coli* native membrane need to be optimized. Briefly, the SMA3000 was added to the well dispersed bacteria membrane suspension, and then the sample was processed with several rounds of sonication-freeze-thaw. Here, negative staining transmission electron microscopy (TEM) was applied to analyze the size of liposomes and vesicles. The *E. coli* native membrane was observed with diameter of about 300 nm (Fig. 2B, left). When the final concentration of 0.15 mmol/L poly-SMA3000 (3:1) was added into liposomes, the diameter of lipodisq was about 100 nm (Fig. 2B, middle). Average diameter of around 10–20 nm (Fig. 2B, right) can be reached, at final concentration of 0.45 mmol/L poly-SMA3000 (3:1). The observed lipodisq with much smaller diameters were consistent with previously reported 10–20 nm size of SMA-lipid nanoparticles (Knowles et al., 2009).

Then, the ^19^F solid state NMR signals were acquired for phosphorylation analysis of ETK-Y574-F_2_Y to verify the improved substrate accessibilities of the membrane lipodisq. Using the previously described mixing procedures of *E. coli* membrane MLV and PTP1B, the trace phosphorylation were removed from the ETK-Y574-F_2_Y sample, and a single ^19^F NMR signal at −133.0 ppm was observed (Fig. 2C), indicating the dephosphorylated F_2_Y (Li et al., 2013). Then, sodium vanadate (Na_3_VO_4_) was added to the sample to suppress the PTP1B function. After addition of Mg^2+^ and ATP to ETK-Y574-F_2_Y, initiating the auto-phosphorylation reaction, the bacteria cell membrane were pellet down using ultracentrifugation for further ^19^F solid state NMR analysis. As shown in Fig. 2D, two ^19^F signals were observed, with one major peak at around −133.2 ppm (dephosphorylated state) and a weak peak around −119.4 ppm (phosphorylated state). This observation was consistent with previously observed small percentage of phosphorylated Y574-F_2_Y in soluble ETK-CD (Li et al., 2013). The observed tyrosine phosphorylation signals of ETK-FL protein in native bacteria membrane were similar as those of *in vitro* analysis of the purified ETK-CD. This verified that the ^19^F-F_2_Y incorporation can be applied for further *in situ* tyrosine phosphorylation analysis of ETK or other membrane proteins.

With the prepared lipodisq of *E. coli* native membrane using SMA300 (3:1), the soluble phosphatase PTP1B or soluble substrate ATP, Mg^2+^ were added to the lipodisq samples directly. Then the samples were pelleted down using ultracentrifugation before solid state NMR analysis. Only one ^19^F signal of dephosphorylated state of ETK-Y574F_2_Y was observed at −133.8 ppm, in the presence of PTP1B (Fig. 2E). One major dephosphorylated signal and one minor phosphorylated signal were observed at −134.1 ppm or −119.8 ppm respectively with the presence of ATP and Mg^2+^ (Fig. 2F). Improved peak sensitivity of the phosphorylated signals (−119.8 ppm) was observed for the lipodisq sample (Fig. 2F) than the liposome sample (Fig. 2D), probably due to the improved accessibility of ATP or Mg^2+^ in the SMA wrapped uni-lamellar liposome and consequent elevated phosphorylation for the ETK-FL- Y574-F_2_Y. Therefore, size homogenous SMA wrap lipodisqs simplified the experiment procedure through improving the substrate accessing to kinase.

Here, we presented the advantage of correlational structure and function studies of membrane proteins in nano-size uni-lamellar lipodisq with SMA surrounding the lipid bilayer, even native cell membrane. Other alternative nano-size uni-lamellar membrane configurations, such as bicelles and nanodiscs, were reported to improve substrate accessibilities to two sides of an incorporated membrane protein in lipid bilayers (Raschle et al., 2010). However, specific lipid, detergents are required for bicelles formation, but resulting in a heterogeneous system (in size and composition) (Raschle et al., 2010). Apo-lipoprotein nanodiscs, composing of a lipid core surrounded by an amphipathic membrane scaffold protein (MSP) (Bayburt and Sligar, 2003, 2010), had the advantage that they could be made from a variety of lipid mixtures, and was relatively mono-dispersed in size upon protein incorporation. Nevertheless, nanodiscs still required detergents for protein incorporation, and could not be directly applied for native membrane system. Moreover, presence of the MSP might bring unpredictable interferences to incorporated proteins or specific interactions to lipids in core. Besides the well integrity maintenance of the native environment and the integral membrane proteins in uni-lamellar lipodisq, the improved accessibility of soluble partner to SMA based lipodisqs in homogenous size provided much convenience for further receptor ligand binding, enzymology analysis and protein-protein interaction analysis in native cell membrane environment using ^19^F solid state NMR or other biophysical methods. Therefore, size homogenous SMA based lipodisqs can not only improve accessibility of soluble partner, but also maintain integrity of the native environment for the integral membrane proteins.

## Electronic supplementary material

Below is the link to the electronic supplementary material.

Supplementary material 1 (PDF 99 kb)
