# Supplementary material for: Nano-size uni-lamellar lipodisq improved in situ auto-phosphorylation analysis of E. coli tyrosine kinase using 19F nuclear magnetic resonance
Source: Protein Cell. 2015 Jan 7;6(3):229–33. doi: 10.1007/s13238-014-0129-x (PMC4348245; doi:10.1007/s13238-014-0129-x)
Supplement: Supplementary file 1 — Supplementary material 1 (PDF 99 kb) [file 13238_2014_129_MOESM1_ESM.pdf]

Nano-size Uni-lamellar Lipodisq Improved *In situ* Auto-phosphorylation Analysis of *E. coli* Tyrosine Kinase using ^{19}F Nuclear Magnetic Resonance

Dong Li[1], Juan Li[1], Yonglong Zhuang[3], Longhua Zhang[1], Ying Xiong[1], Pan Shi[2] *, and Changlin Tian[1,2] *

1. Hefei National Laboratory for Physical Science at the Microscale & School of Life Science, University of Science and Technology of China, Hefei, Anhui 230026, P. R. China

2. High Magnetic Field Laboratory, Chinese Academy of Sciences, Hefei, Anhui 230031, P. R. China

3. Department of Chemistry, Anhui University, Hefei, 230027, P. R. China

1. Plasmids construction and site-specific mutagenesis

A DNA fragment encoding ETK was PCR-amplified from E. coli BL21(DE3), and then it was inserted into p28 vector (with a his-tag in the N terminus) between the Nde I and Xho I restriction endonuclease sites. Tyr574 of ETK was mutated to amber stop codon TAG using a standard PCR-based mutagenesis method. Mutagenesis was confirmed through DNA sequencing. The p28-ETK-Y574TAG plasmid and another important plasmid，pEvol-2FY(containing the coding sequence for tRNACUA and F_2Y-specific aminoacyl-tRNA synthase) were co-transformed in E. coli host cell BL21(DE3)-gold for further protein expression.

2. Full length ETK expression and cell fraction separation

The transformed bacteria were incubated in LB medium containing 0.5 mM F_2Y at 25 ℃. Expression of tRNACUA and F_2Y-specific aminoacyl-tRNA synthase were induced using 0.02% arabinose when the OD600 reached 0.6. Expression of the target protein was induced using 0.8 mM IPTG when the OD600 reached 1.2. After 20 hours，bacteria were harvested using centrifugation at 3724 g for 20 min at 4 ℃. Bacteria pastes were suspended in 40 mL of lysis buffer (50 mM Tris, 300 mM NaCl, pH 8.5). After probe-sonication with a power level of 30% and centrifugation at 17949 g for 30 min at 4 ℃，the pellets containing inclusion bodies, unbroken cells, and insoluble cell debris were discarded. The supernatant was transferred for ultra-centrifuged at 100,000 g, 2 h at 4 ℃. Pellet fraction of the ultracentrifugation was collected for further auto-phosphorylation assay.

3. Liposome preparation and tyrosine auto-phosphorylation assay

The ultra-centrifugation pellets containing bacteria membrane and integral membrane proteins were suspended in buffer (50 mM Tris, 300 mM NaCl, pH 8.5) followed by sonication-freeze-thaw for at least three cycles until well dispersed aliquots. The aliquots were incubated with or without poly-styene-maleic acid (SMA) at 4 ℃ until the clear solution. Total 60 μL SMA were added to the aliquots in three batches. Solutions of liposome with or without SMA were incubated with 2 mM ATP, 1 mM Mg^{2+} or with 1 mg PTP1B (protein tyrosine phosphotase 1b) respectively for 1 hour at room temperature. Activity of PTP1B was suppressed through addition of 10 mM Na3VO4. Finally the solution was ultra-centrifuged at 100,000 g for 2 h at 4 ℃ The pellet was carefully transferred to a 2.5 mm solid state NMR spin rotor.

4. Negative staining transmission electron microscopy (TEM) analysis

After protein expression, bacteria were harvested using centrifugation at 3724 g for 20 min at 4 ℃. Bacteria pastes were suspended in 40 mL of lysis buffer (50 mM Tris, 300 mM NaCl, pH 8.5). After probe-sonication with a power level of 30 % and centrifugation at 17,949 g for 30 min twice at 4℃, the pellet was discarded. Samples of 500 μL supernatant containing 0 μL, 10 μL, 30 μL SMA were prepared (as previously described) for transmission electron microscopy (TEM) analysis. All TEM measurements were carried out on a JEM-2100 high-resolution transmission electron microscopy.

5. ^{19}F solid state NMR data acquisition and processing

All solid state NMR measurements were carried out on a Bruker Avance WB 400 MHz NMR spectrometer at 253 K, equipped with a 2.5 mm broad-band triple-resonance H-F-X MAS probe. The sample spinning rate was 20 kHz controlled by a Bruker pneumatic MAS unit. ^{19}F free induction decays were acquired with 10 k data accumulation with acquisition delay of 4 s. One dimensional ^{19}F spectra were acquired with single pulse (90 pulse width of 3.3 μs), the spectra width of 498.03 ppm and offset at -97.3 ppm. ^{19}F chemical shifts were referenced to trifluoroacetic acid (TFA, 75.39 ppm). The data were analysed and plotted using Topspin 3.1.
